# Parvalbumin-Positive Interneurons Regulate Cortical Sensory Plasticity in Adulthood and Development Through Shared Mechanisms

**DOI:** 10.3389/fncir.2022.886629

**Published:** 2022-05-06

**Authors:** Deborah D. Rupert, Stephen D. Shea

**Affiliations:** ^1^Cold Spring Harbor Laboratory, Cold Spring Harbor, NY, United States; ^2^Medical Scientist Training Program, Stony Brook University, Stony Brook, NY, United States

**Keywords:** parvalbumin, interneurons, plasticity, perineuronal nets, sensory processing, social learning

## Abstract

Parvalbumin-positive neurons are the largest class of GABAergic, inhibitory neurons in the central nervous system. In the cortex, these fast-spiking cells provide feedforward and feedback synaptic inhibition onto a diverse set of cell types, including pyramidal cells, other inhibitory interneurons, and themselves. Cortical inhibitory networks broadly, and cortical parvalbumin-expressing interneurons (cPVins) specifically, are crucial for regulating sensory plasticity during both development and adulthood. Here we review the functional properties of cPVins that enable plasticity in the cortex of adult mammals and the influence of cPVins on sensory activity at four spatiotemporal scales. First, cPVins regulate developmental critical periods and adult plasticity through molecular and structural interactions with the extracellular matrix. Second, they activate in precise sequence following feedforward excitation to enforce strict temporal limits in response to the presentation of sensory stimuli. Third, they implement gain control to normalize sensory inputs and compress the dynamic range of output. Fourth, they synchronize broad network activity patterns in response to behavioral events and state changes. Much of the evidence for the contribution of cPVins to plasticity comes from classic models that rely on sensory deprivation methods to probe experience-dependent changes in the brain. We support investigating naturally occurring, adaptive cortical plasticity to study cPVin circuits in an ethologically relevant framework, and discuss recent insights from our work on maternal experience-induced auditory cortical plasticity.

## Introduction

The cerebral cortex has a remarkable capacity to remodel its synaptic structures and refine its neuronal activity in response to changing conditions throughout life. Such cortical plasticity interacts with a wide range of fundamental biological processes including aging (Freitas et al., 2011), stress (Takatsuru and Koibuchi, 2015; McGirr et al., 2020; Muehlhan et al., 2020), injury and recovery (Grasso et al., 2020; Moreno-López and Hollis, 2021), and sensory learning (LeMessurier and Feldman, 2018). A substantial body of evidence supports the crucial role of GABAergic inhibition in sensory cortical plasticity both during development and adulthood.

The neocortex is built around a “canonical circuit,” a set of vertically connected circuit motifs composed of diverse excitatory and inhibitory cell types that establish common hierarchical neuronal computations (Pluta et al., 2015). This arrangement amplifies thalamic input and integrates that input with projections from other regions to accomplish the complex tasks of sensory processing, learning, and memory. Within the cortex, there is a wide disparity between the numbers of excitatory and inhibitory neurons; glutamatergic excitatory neurons compose the overall majority, whereas GABAergic inhibitory interneurons account for only ∼10–20% of neurons (Markram et al., 2004; Petilla Interneuron Nomenclature Group et al., 2008; DeFelipe et al., 2013; Chu and Anderson, 2015). Despite this mismatch in relative densities, cortical inhibitory drive tends to match excitatory drive (Shadlen and Newsome, 1994; Rubenstein and Merzenich, 2003). This suggests that changes to the synaptic connectivity, morphology, intrinsic properties, and firing patterns of inhibitory neurons play a disproportionate role in controlling the timing and specificity of cortical responses (Hensch, 2004; Lu et al., 2007; Trachtenberg, 2015).

In the last two decades, the contributions of specific inhibitory interneuron subtypes to sensory learning, memory, and plasticity have been studied in the visual, auditory, somatosensory, and olfactory cortices. Here we review some of that literature, focusing on cortical parvalbumin-expressing interneurons (cPVins), which are the most abundant inhibitory cell type in the brain. We discuss how the physiological and molecular features of cPVins equip sensory networks for plasticity. We argue that cPVins across sensory cortices share core intrinsic features and mechanisms that enable periods of experiential learning. We further propose that approaches exploiting ethologically relevant behaviors are important for understanding how cPVin directed plasticity is engaged naturally in the adult brain. While there are many varieties and mechanisms of “plasticity,” our use of the term here denotes adjustments to intrinsic and extrinsic connectivity in sensory cortices that optimize representation of stimuli. We particularly focus on the mechanisms that open and close episodes of heightened sensitivity of cortex to stimuli in response to developmental programs or novel sensory experience.

## Inhibitory Control of Cortical Plasticity

Many of the fundamental features of cortical plasticity were first identified by studies examining the visual cortex during developmental critical periods (e.g., Wiesel and Hubel, 1963; Levay et al., 1978; Gordon and Stryker, 1996; Hensch et al., 1998; Huang et al., 1999; Sawtell et al., 2003). Much of this work observed the consequences of restricting sensory experience, as occurs in monocular deprivation when vision is restricted in one eye. When performed during a specific period early in life, this manipulation weakens responses corresponding to the deprived eye and strengthens responses to the remaining eye, a phenomenon known as ocular dominance plasticity (Hubel and Wiesel, 1963; Fagiolini et al., 1994; Gordon and Stryker, 1996).

The onset and offset of developmental critical periods are defined by changes in cortical GABAergic transmission (Fagiolini et al., 2004; Maffei et al., 2006, 2010). Premature maturation of GABAergic inhibitory networks terminates the V1 developmental critical period precociously (Huang et al., 1999). Mice that are deficient in glutamate decarboxylase (GAD), the rate limiting enzyme involved in synthesis of GABA, have altered neural circuitry and are insensitive to monocular deprivation (Hensch et al., 1998; Fagiolini and Hensch, 2000; Chattopadhyaya et al., 2007). Further, pharmacological disruption of GABA transmission is sufficient to impair typical critical period opening (Jiang et al., 2005; Deidda et al., 2015). On the other hand, complementary approaches that facilitate the development of inhibitory networks accelerate the onset of the critical period (Hanover et al., 1999; Fagiolini and Hensch, 2000; Di Cristo et al., 2007; Sugiyama et al., 2008).

Plasticity continues to be mediated by GABAergic transmission in adulthood (Nahmani and Turrigiano, 2014). The mouse visual cortex retains some plasticity in response to monocular deprivation following critical period closure (Sawtell et al., 2003; Hofer et al., 2006; Fischer et al., 2007; Sato and Stryker, 2008). In the adult V1, cortical potentials corresponding to stimulation of the non-deprived eye are strengthened with experience (Sawtell et al., 2003; Sato and Stryker, 2008). This is associated with enhanced visual acuity in the spared eye (Iny et al., 2006). Manipulations of inhibitory signaling in the adult visual cortex can reactivate experience-dependent plasticity mechanisms. For example, restoring inhibitory function in *GAD* mutants rescues the capacity for plasticity in response to visual deprivation (Hensch et al., 1998; Fagiolini and Hensch, 2000; Iwai et al., 2003; Chattopadhyaya et al., 2007). Interestingly, transplant of inhibitory precursor cells enhances adult cortical plasticity (Southwell et al., 2010; Tang et al., 2014; Davis et al., 2015). On the other hand, Harauzov et al. (2010) reported that reducing intracortical inhibition reactivated visual plasticity, possibly due to a reduction in chondroitin sulfate proteoglycan structures in the extracellular matrix (ECM) called perineuronal nets (PNNs).

Several pieces of early circumstantial evidence implicated cPVins, among all cortical GABAergic populations, as having a central role in regulating visual cortical critical periods. Mutant mice that exhibit an early critical period also show early cPVin maturation (Hanover et al., 1999; Krishnan et al., 2015). Plasticity can be restored in *GAD* mutants by manipulating GABA_*A*_ receptors, which are highly and specifically enriched on cPVins (Fagiolini et al., 2004). More directly, dissolution of cPVin-specific extracellular structures (perineuronal nets- PNNs) reinstates plasticity in adult mice (Pizzorusso et al., 2002). Evidence from Kuhlman et al. (2011, 2013) established that cPVins play a permissive role in critical period plasticity by disinhibiting the excitatory network. However, attempts to reproduce this effect by chemogenetically and optogenetically suppressing cPVin inhibition following monocular deprivation in older mice has yielded mixed results (Kuhlman et al., 2013; Saiepour et al., 2015; Kaplan et al., 2016). This may be due to methodological differences in the parameters used for manipulating PV neurons such as the depth of optical penetration, magnitude, duration, and frequency (El-Boustani and Sur, 2014) or the timing of administration of chemogenetic tools. Interestingly, recent work has shown that monocular deprivation can drastically change the number of synaptic inputs onto V1 cPVins (Ribic, 2020). This suggests that the observed fluctuations in inhibitory drive may be a compensatory mechanism downstream of aberrant synaptic pruning of excitatory inputs onto cPVins (Severin et al., 2021).

## Molecular and Structural Plasticity of Cortical Parvalbumin-Expressing Enterneurons

At the finest spatial scale, cPVin control of cortical plasticity is evident at the level of subcellular interactions with perineuronal structures. The extracellular matrix (ECM) is a complex structure composed of elastin, fibronectin, integrin, glycoprotein, and polysaccharide macromolecules that surrounds cells, and which accounts for 10–20% of the brain’s volume (Cragg, 1979). The ECM supports a wide range of physiological processes including remyelination (You and Gupta, 2018), stem cell storage (Roll and Faissner, 2014), protection against reactive oxygen species (Cabungcal et al., 2013), synapse development (Chan et al., 2020), and stabilization of mature synapses (Frischknecht and Gundelfinger, 2012).

Extracellular matrix components are not uniformly distributed across brain regions or cell types (Dauth et al., 2016). For example, perineuronal nets (PNNs) are chondroitin sulfate glycosaminoglycan (CS-GAG)-based, net-like structures found within ECMs that preferentially surround cPVins and their proximal dendrites (Härtig et al., 1999; Wegner et al., 2003). A hyaluronan backbone acts as a scaffold for assembling CS-GAG, link proteins, and other anchoring proteins into these condensed mesh structures which attach to cell membranes (Brückner et al., 1993; Day and Prestwich, 2002). PNNs contain a number of specific CS-GAGs (e.g., aggrecan, neurocan, brevican) which represent diverse sidechains that attach to lectican protein cores (Giamanco et al., 2010; Frischknecht and Gundelfinger, 2012). How the specific combination of these molecules influences PNN properties is an active area of investigation (Galtrey et al., 2008; Miyata et al., 2018).

Perineuronal nets envelop cPVins and their synapses as developmental windows of plasticity close (Pizzorusso et al., 2002; Berardi et al., 2003; McGee et al., 2005; Nowicka et al., 2009; Carulli et al., 2010; Beurdeley et al., 2012; Ye and Miao, 2013; Happel et al., 2014; Krishnan et al., 2015; Balmer, 2016). This deposition occurs in an activity dependent manner (Reimers et al., 2007; Favuzzi et al., 2017); in fact, cPVin activity is necessary for PNN assembly (Cisneros-Franco and de Villers-Sidani, 2019). The pervasiveness of PNNs fluctuates over the course of an animal’s lifetime in response to sensory experience. These fluctuations are enabled by endogenous metalloproteinase (MMP)— enzymes that degrade the backbone structures of the PNNs (Rossier et al., 2015), and which are themselves regulated by Tissue Inhibitory of Matrix Metalloproteinase (TIMP)-1 (Magnowska et al., 2016). The expression of MMPs declines with the closure of developmental critical periods, but retention of MMPs in the adult brain allows for sensory plasticity through PNN degradation (Murase et al., 2017).

Expression of the parvalbumin (PV) protein itself also fluctuates according to cPVin activity (Kamphuis et al., 1989; Patz et al., 2004). Downregulation of PV has broad consequences for the physiological and synaptic properties of cPVins (Caillard et al., 2000; Schwaller, 2012), as well as for behavior (Wöhr et al., 2015). These effects are likely related to PV’s role in rapidly buffering excess calcium (Aponte et al., 2008). Downregulation of PV expression may also reflect shifts in regional excitatory-inhibitory balance resulting from pathology, for example, as broadly observed in animals models and post-mortem tissue from patients with Autism Spectrum Disorders (Schwaller, 2012; Filice et al., 2016). Moreover, there is evidence of strong mutual influence between the composition and maturity of PNNs and the excitability and activity of cPVins (Balmer, 2016; Favuzzi et al., 2017; Chu et al., 2018; Gottschling et al., 2019; Devienne et al., 2021). The complex relationship between PV expression, cPVin physiology, and PNN assembly are matters of ongoing study, and will further inform our understanding how cPVins regulate cortical plasticity.

Well-developed PNNs appear to be a necessary component for termination of developmental critical periods. Manipulations that disrupt critical period closure (e.g., dark-rearing) delay disinhibition and slow the formation of PNNs (Lander et al., 1997; Pizzorusso et al., 2002). Moreover, genetic knockout of PNN components (Rowland et al., 2021), enzymes required for the synthesis of those components (Hou et al., 2017), or link proteins necessary to establish PNN architecture (Carulli et al., 2010; Ribic, 2020) also prevent critical period closure. Meanwhile, pharmacological inhibition of endogenous MMP activity disrupts ocular dominance plasticity in the adult visual cortex, i.e., a shift in V1 activity to preference the spared eye (Pielecka-Fortuna et al., 2015; Akol et al., 2022).

Conversely, plasticity can be reinstated by pharmacological dissolution of PNNs in the adult cortex, which allows for enhanced synaptic plasticity and physical restructuring of mature PV+ synapses (Magnowska et al., 2016; Ferrer-Ferrer and Dityatev, 2018). For example, in the adult visual cortex, removal of PNNs reinstates most aspects of ocular dominance plasticity, although it is “incomplete” compared to that instated during developmental critical periods (Pizzorusso et al., 2002). This includes disinhibition seen as a drop in V1 cPVin firing rates (Lensjø et al., 2017), and remobilization of dendritic spines, allowing them to lengthen and potentially change the architecture of established circuitry (de Vivo et al., 2013; Faini et al., 2021). Likewise, the effects of monocular deprivation during development on V1 can be partially reversed during adulthood via environmental enrichment, potentially due to a drop in PNNs and reduced inhibitory drive (Sale et al., 2007).

Perineuronal net-fluctuations as a marker of plasticity can also be observed outside of the context of monocular deprivation. For example, reintroduction of light in dark-exposed animals triggers V1 plasticity through the endogenous degradation of the ECM by MMP-9, and resulting disinhibition of fast-spiking interneurons (Murase et al., 2017). In the barrel cortex, whisker trimming leads to downregulation of PV-PNNs (McRae et al., 2007). Finally, evidence from our lab and others also suggests that aberrantly high or low numbers of PNNs during critical periods of sensory learning may impair A1 sound processing (Krishnan et al., 2017; Wen et al., 2018; Pirbhoy et al., 2020).

## Physiological Properties of Cortical Parvalbumin-Expressing Enterneurons

cPVins represent an estimated 5–20% of cells in the brain, and up 40–50% of the inhibitory population, based on developmental progenitor and tracing studies (Tamamaki et al., 2003; Butt et al., 2005; Rudy et al., 2011; Rodarie et al., 2021). Despite similar genetic profiles (Tasic, 2018), and gross numbers of afferent thalamic inputs (Pouchelon et al., 2021) across cortical IN subtypes, individual cPVins have unique cellular physiological characteristics that enable them to locally shape sensory responses.

### Temporal Control of Sensory Responses

One such characteristic is their high spiking rates they exhibited spontaneously *in vivo* or in response to somatic current injection (Connors and Gutnick, 1990), leading to their original label of “fast spiking” cells (Kawaguchi and Hama, 1987; Kawaguchi and Kubota, 1993; Kawaguchi, 1995). “Fast spiking” cells are now widely acknowledged to correspond to PV expressing GABAergic basket cells and to a lesser extent, chandelier cells (Kawaguchi et al., 2019). The fast spiking attributes of cPVins result from their expression of rapidly repolarizing Kv3 potassium channels (Rudy and McBain, 2001) and Ca^2+^-permeable AMPA receptors which have rapid gating kinetics (Jonas et al., 1994; Geiger et al., 1997).

The brisk spiking output and high reliability of cPVin firing enables them to exert tight temporal control over cortical excitation. They convey precisely timed, feedforward inhibition that lags just behind feedforward excitatory input from the thalamus, thereby imposing strict temporal limits (Wehr and Zador, 2003; Zhang et al., 2003; Mittmann et al., 2005; Wilent and Contreras, 2005; Heiss et al., 2009). The potency of this mechanism is increased by cPVins’ particular sensitivity to onsets and offsets of sensory stimuli (Wehr and Zador, 2003; Weible et al., 2014; Keller et al., 2018; Li et al., 2021). Over broader timescales, cPVins make important contributions to stimulus specific adaptation, which allows the brain to maintain sensitivity to novel or behaviorally relevant stimuli among repetitive distractors (Pérez-González and Malmierca, 2012; Natan et al., 2015).

Precise temporal regulation of cortical activity by cPVins is also relevant for more persistent changes in sensory responses through long-term potentiation (LTP) or depression (LTD). These mechanisms rely on coincidence detection of pre- and post- synaptic neuronal spiking. Spike timing-dependent plasticity (STDP), which modifies synapses based on the precise relative timing of individual pre- and post- synaptic spikes (Bi and Poo, 1998), has been widely observed in the cortex (Bender et al., 2006; Meliza and Dan, 2006; Feldman, 2012) and supported by computational models of cortical cell assembles (Wenisch et al., 2005; Klampfl and Maass, 2013). Only more recently has the importance of STDP at inhibitory output synapses, and at cPVin specific synapses, been appreciated (D’Amour and Froemke, 2015; Vickers et al., 2018). Computational modeling predicts that maturation of cPVins helps stabilize STDP such that the most temporally coherent inputs onto cortical pyramidal neurons are strengthened (Kuhlman et al., 2010). Experimental data indicate that LTD of cPVin output onto principal neurons implements cortical disinhibition to regulate plasticity, and is necessary for remodeling the structure of sensory maps (Vickers et al., 2018).

### Selectivity and Gain Control of Sensory Responses

Another important cPVin feature is their broad tuning relative to neighboring pyramidal cells (Runyan et al., 2010; Kuhlman et al., 2011; Li et al., 2012), and other inhibitory subtypes (Kerlin et al., 2010; Ma et al., 2010; Li et al., 2015), however, this observation varies among different sensory brain regions, laminar depth, and among experimentalists (Hofer et al., 2011; Zariwala et al., 2011; Moore and Wehr, 2013).

Many studies have examined the specific operations that cPVins perform on sensory responses through optogenetic activation and/or inactivation of the population. Collectively, these studies have uncovered a panoply of conflicting transformations of sensory representations by cPVins. These include non-selective divisive gain control (Atallah et al., 2012; Wilson et al., 2012; Hamilton et al., 2013), receptive field sharpening (Lee et al., 2012; Kaplan et al., 2016), temporal sharpening (Andermann et al., 2011; Vecchia et al., 2020), non-selective stimulus adaptation (Natan et al., 2015), and spatial constraint of neuronal ensemble activity (Agetsuma et al., 2018).

Broad inhibition of cortical output by cPVins derives from their ability to integrate excitatory activity across a wide area via their extensive dendritic fields (Poo and Isaacson, 2009; Sohal et al., 2009; Packer and Yuste, 2011; Karnani et al., 2014; Hage et al., 2022). This allows cPVins to exert gain control over cortical activity and to maximize the signal-to-noise ratio of information carried to downstream targets. Their intrinsic features also support their synchronized firing (Galarreta and Hestrin, 2001; Bartos et al., 2007; Ferguson and Gao, 2018). First, cPVins are highly sensitive to a large number of high amplitude, phase-locked inputs as a result of their Kv1 channel expression (Goldberg et al., 2008). Second, cPVins are coupled by gap-junctions (Fukuda and Kosaka, 2000; Tamás et al., 2000; Galarreta and Hestrin, 2001; Bartos et al., 2002). Third, cPVins maintain highly branched dendrites to access synaptic input over a sprawling area (Bartholome et al., 2020). In sum, these features facilitate the spread and synchronization of phasic, oscillatory activity across large brain regions.

## cPVin Control of Cortical Network Activity and Behavioral State

At the broadest spatiotemporal scale, cPVin networks have a capacity to coordinate cortical output of large areas. On a population level, the rhythmic firing of cPVins establishes gamma oscillations, a high frequency, (30–80 Hz) periodic signal that results from synchrony of local field potentials and synaptic activity (Buzsáki and Draguhn, 2004). Genetic reduction of the number of cPVins attenuates gamma oscillations (Kalemaki et al., 2018), while optogenetically driving cPVin pools triggers gamma activity (Cardin et al., 2009; Sohal et al., 2009; Etter et al., 2019). Computational models suggest that gamma wave strength directly correlates with the expression of PV and GAD67 (Volman et al., 2011).

Gamma oscillations critically depend on NMDA receptor expression by cPVins (Korotkova et al., 2010; Gonzalez-Burgos and Lewis, 2012). In contrast to the fast spiking properties of cPVins, NMDA receptors activate synaptic currents with slow kinetics (Forsythe and Westbrook, 1988). The key contribution of NMDA receptors to the establishment of gamma rhythms appears to be their capacity to stabilize and coordinate recruitment of cPVins into synchronous ensembles (Carlén et al., 2012; Cornford et al., 2019).

The functional roles of gamma oscillations are less well understood. Oscillations reflect cPVin task-dependent activity and recruitment of greater numbers of cPVins during sensory learning (Gotts et al., 2012; Brunet et al., 2014; Ainsworth et al., 2016). Changes in gamma activity are critical for learning rules, such as during operant conditioning (Caroni, 2015; Lintas et al., 2021). Moreover, they are necessary for updating cortical responses when there is a mismatch between the history of stimuli-salience association and the new rules of the rules of sensory learning (Cho et al., 2020).

Further, the presence of gamma oscillations also correlates with the familiarity of sensory stimuli, which suggests they facilitate memory traces in sensory cortices (Womelsdorf et al., 2006; Headley and Weinberger, 2011; Weinberger et al., 2013; Cooke et al., 2015; Kissinger et al., 2018, 2020). In addition these oscillations may contribute to storing memories (Galuske et al., 2019). Gamma waves can also be triggered in response to meaningful behavioral outcomes (Fries et al., 2001; Kim et al., 2015; Ray and Maunsell, 2015; Cardin, 2016; Cho et al., 2020), which could imply their contribution to salience encoding.

Finally, gamma power in sensory cortices correlates with behavioral states, such as attention (Börgers et al., 2008; Sobolewski et al., 2011; Bosman et al., 2012; Kim et al., 2016), locomotion (Niell and Stryker, 2010), and arousal (Kim et al., 2015; Vinck et al., 2015). Evidence to date suggests that gamma rhythms in sensory cortex reflect top-down, modulated cPVin activity that integrates learning and memory contexts.

## cPVin Control of Maternal-Experience Induced Auditory Cortical Plasticity

In mice, plasticity in the auditory cortex (ACtx) is triggered by maternal experience with pups. A broad base of evidence supports the conclusion that auditory plasticity in adult females helps to sharpen and amplify responses to behaviorally salient distress vocalizations from the pups. Here we review these data, highlighting work by our lab identifying cPVin-specific physiological mechanisms underlying “maternal,” experience-dependent auditory plasticity. These mechanisms share features of classical models of experience-dependent cortical plasticity, including those observed in developmental critical periods. Importantly, however, this plasticity is activated not by an exogenous experimental manipulation, but simply by social experience.

Primiparous mice learn to recognize and respond to pup ultrasonic vocalizations (USVs), which signal distress when pups are isolated and become hypothermic. This learning is manifested behaviorally in the retrieval of pups back to the nest by the dams (Sewell, 1970; Ehret et al., 1987). However, the same learning is also exhibited by co-housed, virgin females (“surrogates”) outside of the influence of pregnancy-induced hormone fluctuations (Rosenblatt, 1967; Galindo-Leon et al., 2009; Cohen et al., 2011; Cohen and Mizrahi, 2015; Stolzenberg and Champagne, 2016; Krishnan et al., 2017; Lau et al., 2020; Carcea et al., 2021). Importantly, retrieval learning correlates with plasticity of ACtx inhibitory networks (Liu and Schreiner, 2007; Galindo-Leon et al., 2009; Cohen et al., 2011; Lin et al., 2013; Cohen and Mizrahi, 2015; Marlin et al., 2015; Lau et al., 2020). Specifically, ACtx pyramidal cells from pup or ultrasonic vocalization exposed females demonstrate time-locked responses. However, these responses are attenuated in naïve counterparts.

Our mechanistic understanding of maternal experience-induced plasticity has been enhanced by our research with *Mecp2* mutant mouse models. While wild-type (WT) maternal surrogates readily learn to perform pup retrieval, adult female mice that are missing one copy of the X chromosome -linked *Mecp2* gene (*Mecp2^HET^*) fail to perform this task (Krishnan et al., 2017). We examined potential molecular mechanisms underlying this behavioral phenotype and found that exposure to pups initiated a transient increase in expression of GAD67 in the ACtx of both *Mecp2^HET^* subjects and wild type littermates (*Mecp2^WT^*) (Krishnan et al., 2017). However, pup experience also leads to overexpression of parvalbumin protein (PV) and cPVin-associated PNNs in the ACtx of *Mecp2^HET^* subjects, relative to naïve and *Mecp2^WT^* controls (Krishnan et al., 2017). In the context of the literature discussed above, we hypothesized that the overexpression of PV and PNNs in the brains of *Mecp2^HET^* surrogates inhibits the ACtx plasticity underlying retrieval learning, paralleling restriction of developmental critical periods. That is, while depression of these markers was not observed in WT subjects, their aberrant elevation in *Mecp2^HET^* surrogates is sufficient for impairing behavioral responses to USVs. Therefore we surmise that a threshold level of PV and PNN expression, below which ACtx cells of *Mecp2^WT^* controls maintain expression, allows for normal learning.

Further, we predicted that dissolution of PNNs might reinstate ACtx plasticity, as has been found in other sensory cortices. Indeed, PNN dissolution in the ACtx just prior to birth of the pups ameliorated overexpression of PNNs and facilitated retrieval performance in *Mecp2^HET^* subjects (Krishnan et al., 2017). Again this suggests a cPVin specific mechanism or disruption that underlies the ability of *Mecp2^HET^* subjects to respond behaviorally to USV cues. We further linked cPVins to maternal learning by showing that deletion of Mecp2 in cPVins was sufficient to disrupt early retrieval (Krishnan et al., 2017).

We next examined the effects of pup exposure on stimulus-driven activity in the ACtx of awake mice. Consistent with a central role of cPVin inhibitory drive in the pup-induced ACtx plasticity, we found that, in *Mecp2^WT^* subjects, responses of cPVins to USVs were weaker after experience with pups (Lau et al., 2020). The diminished output of individual cPVins was mirrored by a complementary increase in the magnitude of responses from excitatory, putative pyramidal ACtx neurons (Lau et al., 2020). That is, maternal experience triggers a drop in cPVin responses to USV playback in the ACtx of *Mecp2^WT^*. This is complemented by increased pyramidal neuron responses to USVs.

In contrast, these physiological changes were not observed in the ACtx of *Mecp2^Het^*. More specifically, cPVin activity in response to USVs remained high despite experience with pups. These firing patterns matched the observed elevation in PV protein expression in *Mecp2^Het^*, but not *Mecp2^WT^* subjects. That is, given the activity-dependent expression of PV, the continued high stimulus-evoked firing rates of cPVins in *Mecp2^Het^* links the cPVin-specific molecular expression to a functional lack of plasticity.

This pattern of results is also consistent with our understanding of sensory cortical plasticity in other systems, which is often initiated by disinhibition. In sum, inhibitory ACtx plasticity maximizes neural “contrast” and appears to align temporal dynamics of cortical response to USVs, enabling pup retrieval (Liu and Schreiner, 2007; Shepard et al., 2015). These findings are very much in keeping with the physiological properties of cPVin that allow them to rapidly regulate the timing of cortical output.

As others have suggested, studying sensory processing in natural and/or socially salient contexts may be particularly effective at triggering rapid or robust plasticity (Taborsky et al., 2015). Our work uses a maternal experience paradigm to evoke a natural and spontaneous form of cPVin-mediated sensory cortical plasticity in adult animals. However, there are many remaining questions to be answered. What does the population level structure of cPVin activity, including cortical gamma oscillatory activity, in response to USV calls look like, and how does experience with pups change those patterns? Do large scale oscillations reflect, or regulate, synchronous activity that favors synaptic modification in response to behavioral state? How does the activity of cPVins, and other inhibitory cortical populations, differ in freely behaving mice, as opposed to awake, head-fixed mice? What is the contribution, if any, of other cortical inhibitory subpopulations to maternal experience-induced auditory plasticity? How is cPVin-mediated, sensory cortical plasticity modulated by multisensory integration? And how might it be modulated by behavioral states including attention, arousal, and emotional salience or valence?

Here we briefly summarize the shared features of plasticity during visual, developmental critical periods and during adult, auditory learning in natural contexts. First, both are marked by dynamic downregulation of cortical inhibition that results from decreased stimulus-evoked firing by cPVins, and leads to a concomitant increase in excitability of principal cortical neurons. This likely reflects changes to relative synaptic strengths from cPVins that lower the LTP threshold of pyramidal cells (Smith et al., 2009). Second, this cortical disinhibition is permissive for changes in connectivity, including the number of inputs by cPVins onto pyramidal cells. Third, periods of plasticity are delineated by fluctuations in the expression of parvalbumin protein and perineuronal nets, including prominent elevation of these markers as windows of heightened plasticity close. These molecular fluctuations may aid in mobility, or pruning, of synaptic inputs (Huang et al., 2015) that facilitate synaptic plasticity. Finally, in both cases, changes that reflect cortical plasticity do not preclude mechanisms of plasticity upstream of the cortex, e.g., changes to thalamic projections onto cPVins spurred by sensory experiences (Sommeijer et al., 2017). Nevertheless, the similarity in cortical plasticity features provides a general framework through which we can examine cPVin-specific contributions to sensory learning.

## Conclusion and Future Directions

Here we have focused on the known contributions of functional and structural cPVin plasticity for supporting sensory learning. However, the current understanding is far from complete. There are many areas of on-going investigation which promise to enhance this understanding including: contributions of cPVin to encoding natural or other complex stimuli (Zhu et al., 2015), real-time dynamics between cPVins and other cell types that make up canonical units (Rikhye et al., 2021), modulation of cPVins by top-down brain-state dynamics (Pakan et al., 2016), better dissection of transcriptomic (Fishell and Kepecs, 2020) and spatial (Large et al., 2018) profiles, and cPVin circuit specificity (Jiang et al., 2021).

We have also emphasized, by highlighting our own work, the need for further dissection of cell type specific plasticity in naturally occurring, socially salient contexts. Technical advancements, such as the development of less invasive approaches, are particularly important for implementing such investigations. For example, extracranial delivery of optogenetic (Chen et al., 2021) and ultrasonic stimulations (Estrada et al., 2021) as well as wireless recording capabilities (Shin et al., 2022) will make observation and manipulation of cell type specific populations during freely moving task engagement less cumbersome.

Advancing our understanding of cPVin plasticity has implications beyond scientific inquiry. Aberrant cPVin circuitry has been implicated in a diverse set of neurocognitive conditions (Huang, 2014; Ferguson and Gao, 2018; Filice et al., 2020). Therefore, a thorough understanding of cell type specific contributions to synaptic plasticity and network-wide oscillatory patterns has wide-spread implications for diagnostic and therapeutic advancement.

## Author Contributions

Both authors listed have made a substantial, direct, and intellectual contribution to the work, and approved it for publication.

## Conflict of Interest

The authors declare that the research was conducted in the absence of any commercial or financial relationships that could be construed as a potential conflict of interest.

## Publisher’s Note

All claims expressed in this article are solely those of the authors and do not necessarily represent those of their affiliated organizations, or those of the publisher, the editors and the reviewers. Any product that may be evaluated in this article, or claim that may be made by its manufacturer, is not guaranteed or endorsed by the publisher.

## References

[B1] Agetsuma M. Hamm J. P. Tao K. Fujisawa S. Yuste R. (2018). Parvalbumin-positive interneurons regulate neuronal ensembles in visual cortex. *Cereb. Cortex* 28 1831–1845. 10.1093/cercor/bhx169 29106504 PMC5907345

[B2] Ainsworth M. Lee S. Kaiser M. Simonotto J. Kopell N. J. Whittington M. A. (2016). GABAB receptor-mediated, layer-specific synaptic plasticity reorganizes gamma-frequency neocortical response to stimulation. *Proc. Natl. Acad. Sci. U.S.A.* 113 E2721–E2729. 10.1073/pnas.1605243113 27118845 PMC4868473

[B3] Akol I. Kalogeraki E. Pielecka-Fortuna J. Fricke M. Löwel S. (2022). MMP2 and MMP9 activity is crucial for adult visual cortex plasticity in healthy and stroke-affected mice. *J. Neurosci.* 42 16–32. 10.1523/JNEUROSCI.0902-21.2021 34764155 PMC8741160

[B4] Andermann M. L. Kerlin A. M. Roumis D. K. Glickfeld L. L. Reid R. C. (2011). Functional specialization of mouse higher visual cortical areas. *Neuron* 72 1025–1039. 10.1016/j.neuron.2011.11.013 22196337 PMC3876958

[B5] Aponte Y. Bischofberger J. Jonas P. (2008). Efficient Ca^2+^ buffering in fast-spiking basket cells of rat hippocampus. *J. Physiol.* 586 2061–2075. 10.1113/jphysiol.2007.147298 18276734 PMC2465201

[B6] Atallah B. V. Bruns W. Carandini M. Scanziani M. (2012). Parvalbumin-expressing interneurons linearly transform cortical responses to visual stimuli. *Neuron* 73 159–170. 10.1016/j.neuron.2011.12.013 22243754 PMC3743079

[B7] Balmer T. S. (2016). Perineuronal nets enhance the excitability of fast-spiking neurons. *eNeuro* 3:ENEURO.0112-16.2016. 10.1523/ENEURO.0112-16.2016 27570824 PMC4987413

[B8] Bartholome O. de la Brassinne Bonardeaux O. Neirinckx V. Rogister B. (2020). A composite sketch of fast-spiking parvalbumin-positive neurons. *Cereb. Cortex Commun.* 1:tgaa026. 10.1093/texcom/tgaa026 34296100 PMC8153048

[B9] Bartos M. Vida I. Frotscher M. Meyer A. Monyer H. Geiger J. R. P. (2002). Fast synaptic inhibition promotes synchronized gamma oscillations in hippocampal interneuron networks. *Proc. Natl. Acad. Sci. U.S.A.* 99 13222–13227. 10.1073/pnas.192233099 12235359 PMC130614

[B10] Bartos M. Vida I. Jonas P. (2007). Synaptic mechanisms of synchronized gamma oscillations in inhibitory interneuron networks. *Nat. Rev. Neurosci.* 8 45–56. 10.1038/nrn2044 17180162

[B11] Bender V. A. Bender K. J. Brasier D. J. Feldman D. E. (2006). Two coincidence detectors for spike timing-dependent plasticity in somatosensory cortex. *J. Neurosci.* 26 4166–4177. 10.1523/JNEUROSCI.0176-06.2006 16624937 PMC3071735

[B12] Berardi N. Pizzorusso T. Ratto G. M. Maffei L. (2003). Molecular basis of plasticity in the visual cortex. *Trends Neurosci.* 26 369–378. 10.1016/S0166-2236(03)00168-1 12850433

[B13] Beurdeley M. Spatazza J. Lee H. H. C. Sugiyama S. Bernard C. Nardo A. A. D. (2012). Otx2 binding to perineuronal nets persistently regulates plasticity in the mature visual cortex. *J. Neurosci.* 32 9429–9437. 10.1523/JNEUROSCI.0394-12.2012 22764251 PMC3419577

[B14] Bi G. Poo M. (1998). Synaptic modifications in cultured hippocampal neurons: dependence on spike timing, synaptic strength, and postsynaptic cell type. *J. Neurosci.* 18 10464–10472. 10.1523/JNEUROSCI.18-24-10464.1998 9852584 PMC6793365

[B15] Börgers C. Epstein S. Kopell N. J. (2008). Gamma oscillations mediate stimulus competition and attentional selection in a cortical network model. *Proc. Natl. Acad. Sci. U.S.A.* 105 18023–18028. 10.1073/pnas.0809511105 19004759 PMC2584712

[B16] Bosman C. A. Schoffelen J.-M. Brunet N. Oostenveld R. Bastos A. M. Womelsdorf T. (2012). Attentional stimulus selection through selective synchronization between monkey visual areas. *Neuron* 75 875–888. 10.1016/j.neuron.2012.06.037 22958827 PMC3457649

[B17] Brückner G. Brauer K. Härtig W. Wolff J. R. Rickmann M. J. Derouiche A. (1993). Perineuronal nets provide a polyanionic, glia-associated form of microenvironment around certain neurons in many parts of the rat brain. *Glia* 8 183–200. 10.1002/glia.440080306 7693589

[B18] Brunet N. M. Bosman C. A. Vinck M. Roberts M. Oostenveld R. Desimone R. (2014). Stimulus repetition modulates gamma-band synchronization in primate visual cortex. *Proc. Natl. Acad. Sci. U.S.A.* 111 3626–3631. 10.1073/pnas.1309714111 24554080 PMC3948273

[B19] Butt S. J. B. Fuccillo M. Nery S. Noctor S. Kriegstein A. Corbin J. G. (2005). The temporal and spatial origins of cortical interneurons predict their physiological subtype. *Neuron* 48 591–604. 10.1016/j.neuron.2005.09.034 16301176

[B20] Buzsáki G. Draguhn A. (2004). Neuronal oscillations in cortical networks. *Science* 304 1926–1929. 10.1126/science.1099745 15218136

[B21] Cabungcal J.-H. Steullet P. Morishita H. Kraftsik R. Cuenod M. Hensch T. K. (2013). Perineuronal nets protect fast-spiking interneurons against oxidative stress. *Proc. Natl. Acad. Sci. U.S.A.* 110 9130–9135. 10.1073/pnas.1300454110 23671099 PMC3670388

[B22] Caillard O. Moreno H. Schwaller B. Llano I. Celio M. R. Marty A. (2000). Role of the calcium-binding protein parvalbumin in short-term synaptic plasticity. *Proc. Natl. Acad. Sci. U.S.A.* 97 13372–13377. 10.1073/pnas.230362997 11069288 PMC27231

[B23] Carcea I. Caraballo N. L. Marlin B. J. Ooyama R. Riceberg J. S. Mendoza Navarro J. M. (2021). Oxytocin neurons enable social transmission of maternal behaviour. *Nature* 596 553–557. 10.1038/s41586-021-03814-7 34381215 PMC8387235

[B24] Cardin J. A. (2016). Snapshots of the brain in action: local circuit operations through the lens of oscillations. *J. Neurosci.* 36 10496–10504. 10.1523/JNEUROSCI.1021-16.2016 27733601 PMC5059425

[B25] Cardin J. A. Carlén M. Meletis K. Knoblich U. Zhang F. Deisseroth K. (2009). Driving fast-spiking cells induces gamma rhythm and controls sensory responses. *Nature* 459 663–667. 10.1038/nature08002 19396156 PMC3655711

[B26] Carlén M. Meletis K. Siegle J. H. Cardin J. A. Futai K. Vierling-Claassen D. (2012). A critical role for NMDA receptors in parvalbumin interneurons for gamma rhythm induction and behavior. *Mol. Psychiatry* 17 537–548. 10.1038/mp.2011.31 21468034 PMC3335079

[B27] Caroni P. (2015). Regulation of parvalbumin basket cell plasticity in rule learning. *Biochem. Biophys. Res. Commun.* 460 100–103. 10.1016/j.bbrc.2015.02.023 25998738

[B28] Carulli D. Pizzorusso T. Kwok J. C. F. Putignano E. Poli A. Forostyak S. (2010). Animals lacking link protein have attenuated perineuronal nets and persistent plasticity. *Brain* 133 2331–2347. 10.1093/brain/awq145 20566484

[B29] Chan Z. C.-K. Oentaryo M. J. Lee C. W. (2020). MMP-mediated modulation of ECM environment during axonal growth and NMJ development. *Neurosci. Lett.* 724:134822. 10.1016/j.neulet.2020.134822 32061716

[B30] Chattopadhyaya B. Di Cristo G. Wu C. Z. Knott G. Kuhlman S. Fu Y. (2007). GAD67-mediated GABA synthesis and signaling regulate inhibitory synaptic innervation in the visual cortex. *Neuron* 54 889–903. 10.1016/j.neuron.2007.05.015 17582330 PMC2077924

[B31] Chen R. Gore F. Nguyen Q.-A. Ramakrishnan C. Patel S. Kim S. H. (2021). Deep brain optogenetics without intracranial surgery. *Nat. Biotechnol.* 39 161–164. 10.1038/s41587-020-0679-9 33020604 PMC7878426

[B32] Cho K. K. A. Davidson T. J. Bouvier G. Marshall J. D. Schnitzer M. J. Sohal V. S. (2020). Cross-hemispheric gamma synchrony between prefrontal parvalbumin interneurons supports behavioral adaptation during rule shift learning. *Nat. Neurosci.* 23 892–902. 10.1038/s41593-020-0647-1 32451483 PMC7347248

[B33] Chu J. Anderson S. A. (2015). Development of cortical interneurons. *Neuropsychopharmacology* 40 16–23. 10.1038/npp.2014.171 25103177 PMC4262895

[B34] Chu P. Abraham R. Budhu K. Khan U. De Marco Garcia N. Brumberg J. C. (2018). The impact of perineuronal net digestion using chondroitinase ABC on the intrinsic physiology of cortical neurons. *Neuroscience* 388 23–35. 10.1016/j.neuroscience.2018.07.004 30004010 PMC6338339

[B35] Cisneros-Franco J. M. de Villers-Sidani É (2019). Reactivation of critical period plasticity in adult auditory cortex through chemogenetic silencing of parvalbumin-positive interneurons. *Proc. Natl. Acad. Sci. U.S.A.* 116 26329–26331. 10.1073/pnas.1913227117 31843881 PMC6936688

[B36] Cohen L. Mizrahi A. (2015). Plasticity during motherhood: changes in excitatory and inhibitory layer 2/3 neurons in auditory cortex. *J. Neurosci.* 35 1806–1815. 10.1523/JNEUROSCI.1786-14.2015 25632153 PMC6795264

[B37] Cohen L. Rothschild G. Mizrahi A. (2011). Multisensory integration of natural odors and sounds in the auditory cortex. *Neuron* 72 357–369. 10.1016/j.neuron.2011.08.019 22017993

[B38] Connors B. W. Gutnick M. J. (1990). Intrinsic firing patterns of diverse neocortical neurons. *Trends Neurosci.* 13, 99–104. 10.1016/0166-2236(90)90185-D 1691879

[B39] Cooke S. F. Komorowski R. W. Kaplan E. S. Gavornik J. P. Bear M. F. (2015). Visual recognition memory, manifested as long-term habituation, requires synaptic plasticity in V1. *Nat. Neurosci.* 18 262–271. 10.1038/nn.3920 25599221 PMC4383092

[B40] Cornford J. H. Mercier M. S. Leite M. Magloire V. Häusser M. Kullmann D. M. (2019). Dendritic NMDA receptors in parvalbumin neurons enable strong and stable neuronal assemblies. *eLife* 8:e49872. 10.7554/eLife.49872 31657720 PMC6839945

[B41] Cragg B. (1979). Brain extracellular space fixed for electron microscopy. *Neurosci. Lett.* 15 301–306. 10.1016/0304-3940(79)96130-5 394033

[B42] D’Amour J. A. Froemke R. C. (2015). Inhibitory and excitatory spike-timing-dependent plasticity in the auditory cortex. *Neuron* 86 514–528. 10.1016/j.neuron.2015.03.014 25843405 PMC4409545

[B43] Dauth S. Grevesse T. Pantazopoulos H. Campbell P. H. Maoz B. M. Berretta S. (2016). Extracellular matrix protein expression is brain region dependent. *J. Comp. Neurol.* 524 1309–1336. 10.1002/cne.23965 26780384 PMC7714387

[B44] Davis M. F. Figueroa Velez D. X. Guevarra R. P. Yang M. C. Habeeb M. Carathedathu M. C. (2015). Inhibitory neuron transplantation into adult visual cortex creates a new critical period that rescues impaired vision. *Neuron* 86 1055–1066. 10.1016/j.neuron.2015.03.062 25937171 PMC4441572

[B45] Day A. J. Prestwich G. D. (2002). Hyaluronan-binding proteins: tying up the giant. *J. Biol. Chem.* 277 4585–4588. 10.1074/jbc.R100036200 11717315

[B46] de Vivo L. Landi S. Panniello M. Baroncelli L. Chierzi S. Mariotti L. (2013). Extracellular matrix inhibits structural and functional plasticity of dendritic spines in the adult visual cortex. *Nat. Commun.* 4:1484. 10.1038/ncomms2491 23403561

[B47] DeFelipe J. López-Cruz P. L. Benavides-Piccione R. Bielza C. Larrañaga P. Anderson S. (2013). New insights into the classification and nomenclature of cortical GABAergic interneurons. *Nat. Rev. Neurosci.* 14 202–216. 10.1038/nrn3444 23385869 PMC3619199

[B48] Deidda G. Allegra M. Cerri C. Naskar S. Bony G. Zunino G. (2015). Early depolarizing GABA controls critical period plasticity in the rat visual cortex. *Nat. Neurosci.* 18 87–96. 10.1038/nn.3890 25485756 PMC4338533

[B49] Devienne G. Picaud S. Cohen I. Piquet J. Tricoire L. Testa D. (2021). Regulation of perineuronal nets in the adult cortex by the activity of the cortical network. *J. Neurosci.* 41 5779–5790. 10.1523/JNEUROSCI.0434-21.2021 34045309 PMC8265812

[B50] Di Cristo G. Chattopadhyaya B. Kuhlman S. J. Fu Y. Bélanger M.-C. Wu C. Z. (2007). Activity-dependent PSA expression regulates inhibitory maturation and onset of critical period plasticity. *Nat. Neurosci.* 10 1569–1577. 10.1038/nn2008 18026099

[B51] Ehret G. Koch M. Haack B. Markl H. (1987). Sex and parental experience determine the onset of an instinctive behavior in mice. *Naturwissenschaften* 74:47. 10.1007/BF00367047 3561521

[B52] El-Boustani S. Sur M. (2014). Response-dependent dynamics of cell-specific inhibition in cortical networks in vivo. *Nat. Commun.* 5:5689. 10.1038/ncomms6689 25504329 PMC4268659

[B53] Estrada H. Robin J. Özbek A. Chen Z. Marowsky A. Zhou Q. (2021). High-resolution fluorescence-guided transcranial ultrasound mapping in the live mouse brain. *Sci. Adv.* 7:eabi5464. 10.1126/sciadv.abi5464 34878843 PMC8654306

[B54] Etter G. van der Veldt S. Manseau F. Zarrinkoub I. Trillaud-Doppia E. Williams S. (2019). Optogenetic gamma stimulation rescues memory impairments in an Alzheimer’s disease mouse model. *Nat. Commun.* 10:5322. 10.1038/s41467-019-13260-9 31757962 PMC6876640

[B55] Fagiolini M. Fritschy J.-M. Löw K. Möhler H. Rudolph U. Hensch T. K. (2004). Specific GABAA circuits for visual cortical plasticity. *Science* 303 1681–1683. 10.1126/science.1091032 15017002

[B56] Fagiolini M. Hensch T. K. (2000). Inhibitory threshold for critical-period activation in primary visual cortex. *Nature* 404 183–187. 10.1038/35004582 10724170

[B57] Fagiolini M. Pizzorusso T. Berardi N. Domenici L. Maffei L. (1994). Functional postnatal development of the rat primary visual cortex and the role of visual experience: dark rearing and monocular deprivation. *Vision Res.* 34 709–720. 10.1016/0042-6989(94)90210-0 8160387

[B58] Faini G. Del Bene F. Albadri S. (2021). Reelin functions beyond neuronal migration: from synaptogenesis to network activity modulation. *Curr. Opin. Neurobiol.* 66 135–143. 10.1016/j.conb.2020.10.009 33197872

[B59] Favuzzi E. Marques-Smith A. Deogracias R. Winterflood C. M. Sánchez-Aguilera A. Mantoan L. (2017). Activity-dependent gating of parvalbumin interneuron function by the perineuronal net protein brevican. *Neuron* 95 639–655.e10. 10.1016/j.neuron.2017.06.028 28712654

[B60] Feldman D. E. (2012). The spike timing dependence of plasticity. *Neuron* 75 556–571. 10.1016/j.neuron.2012.08.001 22920249 PMC3431193

[B61] Ferguson B. R. Gao W.-J. (2018). Pv interneurons: critical regulators of E/I balance for prefrontal cortex-dependent behavior and psychiatric disorders. *Front. Neural Circuits* 12:37. 10.3389/fncir.2018.00037 29867371 PMC5964203

[B62] Ferrer-Ferrer M. Dityatev A. (2018). Shaping synapses by the neural extracellular matrix. *Front. Neuroanat.* 12:40. 10.3389/fnana.2018.00040 29867379 PMC5962695

[B63] Filice F. Janickova L. Henzi T. Bilella A. Schwaller B. (2020). The parvalbumin hypothesis of autism spectrum disorder. *Front. Cell. Neurosci.* 14:577525. 10.3389/fncel.2020.577525 33390904 PMC7775315

[B64] Filice F. Vörckel K. J. Sungur A. Ö Wöhr M. Schwaller B. (2016). Reduction in parvalbumin expression not loss of the parvalbumin-expressing GABA interneuron subpopulation in genetic parvalbumin and shank mouse models of autism. *Mol. Brain* 9:10. 10.1186/s13041-016-0192-8 26819149 PMC4729132

[B65] Fischer Q. S. Aleem S. Zhou H. Pham T. A. (2007). Adult visual experience promotes recovery of primary visual cortex from long-term monocular deprivation. *Learn. Mem.* 14 573–580. 10.1101/lm.676707 17761542 PMC1994076

[B66] Fishell G. Kepecs A. (2020). Interneuron types as attractors and controllers. *Annu. Rev. Neurosci.* 43 1–30. 10.1146/annurev-neuro-070918-050421 31299170 PMC7064158

[B67] Forsythe I. D. Westbrook G. L. (1988). Slow excitatory postsynaptic currents mediated by N-methyl-D-aspartate receptors on cultured mouse central neurones. *J. Physiol.* 396 515–533. 10.1113/jphysiol.1988.sp016975 2900892 PMC1192058

[B68] Freitas C. Perez J. Knobel M. Tormos J. M. Oberman L. Eldaief M. (2011). Changes in cortical plasticity across the lifespan. *Front. Aging Neurosci.* 3:5. 10.3389/fnagi.2011.00005 21519394 PMC3079175

[B69] Fries P. Reynolds J. H. Rorie A. E. Desimone R. (2001). Modulation of oscillatory neuronal synchronization by selective visual attention. *Science* 291 1560–1563. 10.1126/science.1055465 11222864

[B70] Frischknecht R. Gundelfinger E. D. (2012). The brain’s extracellular matrix and its role in synaptic plasticity. *Adv. Exp. Med. Biol.* 970 153–171. 10.1007/978-3-7091-0932-8_7 22351055

[B71] Fukuda T. Kosaka T. (2000). Gap junctions linking the dendritic network of GABAergic interneurons in the hippocampus. *J. Neurosci.* 20 1519–1528. 10.1523/JNEUROSCI.20-04-01519.2000 10662841 PMC6772381

[B72] Galarreta M. Hestrin S. (2001). Spike transmission and synchrony detection in networks of GABAergic interneurons. *Science* 292 2295–2299. 10.1126/science.1061395 11423653

[B73] Galindo-Leon E. E. Lin F. G. Liu R. C. (2009). Inhibitory plasticity in a lateral band improves cortical detection of natural vocalizations. *Neuron* 62 705–716. 10.1016/j.neuron.2009.05.001 19524529 PMC2709999

[B74] Galtrey C. M. Kwok J. C. F. Carulli D. Rhodes K. E. Fawcett J. W. (2008). Distribution and synthesis of extracellular matrix proteoglycans, hyaluronan, link proteins and tenascin-R in the rat spinal cord. *Eur. J. Neurosci.* 27 1373–1390. 10.1111/j.1460-9568.2008.06108.x 18364019

[B75] Galuske R. A. W. Munk M. H. J. Singer W. (2019). Relation between gamma oscillations and neuronal plasticity in the visual cortex. *Proc. Natl. Acad. Sci. U.S.A.* 116 23317–23325. 10.1073/pnas.1901277116 31659040 PMC6859324

[B76] Geiger J. R. P. Lübke J. Roth A. Frotscher M. Jonas P. (1997). Submillisecond ampa receptor-mediated signaling at a principal neuron–interneuron synapse. *Neuron* 18 1009–1023. 10.1016/S0896-6273(00)80339-6 9208867

[B77] Giamanco K. A. Morawski M. Matthews R. T. (2010). Perineuronal net formation and structure in aggrecan knockout mice. *Neuroscience* 170 1314–1327. 10.1016/j.neuroscience.2010.08.032 20732394

[B78] Goldberg E. M. Clark B. D. Zagha E. Nahmani M. Erisir A. Rudy B. (2008). K^+^ channels at the axon initial segment dampen near-threshold excitability of neocortical fast-spiking GABAergic interneurons. *Neuron* 58 387–400. 10.1016/j.neuron.2008.03.003 18466749 PMC2730466

[B79] Gonzalez-Burgos G. Lewis D. A. (2012). NMDA receptor hypofunction, parvalbumin-positive neurons, and cortical gamma oscillations in schizophrenia. *Schizophr. Bull.* 38 950–957. 10.1093/schbul/sbs010 22355184 PMC3446219

[B80] Gordon J. A. Stryker M. P. (1996). Experience-dependent plasticity of binocular responses in the primary visual cortex of the mouse. *J. Neurosci.* 16 3274–3286. 10.1523/JNEUROSCI.16-10-03274.1996 8627365 PMC6579137

[B81] Gotts S. J. Chow C. C. Martin A. (2012). Repetition priming and repetition suppression: a case for enhanced efficiency through neural synchronization. *Cogn. Neurosci.* 3 227–237. 10.1080/17588928.2012.670617 23144664 PMC3491809

[B82] Gottschling C. Wegrzyn D. Denecke B. Faissner A. (2019). Elimination of the four extracellular matrix molecules tenascin-C, tenascin-R, brevican and neurocan alters the ratio of excitatory and inhibitory synapses. *Sci. Rep.* 9:13939. 10.1038/s41598-019-50404-9 31558805 PMC6763627

[B83] Grasso P. A. Gallina J. Bertini C. (2020). Shaping the visual system: cortical and subcortical plasticity in the intact and the lesioned brain. *Neuropsychologia* 142:107464. 10.1016/j.neuropsychologia.2020.107464 32289349

[B84] Hage T. A. Bosma-Moody A. Baker C. A. Kratz M. B. Campagnola L. Jarsky T. (2022). Synaptic connectivity to L2/3 of primary visual cortex measured by two-photon optogenetic stimulation. *eLife* 11:e71103. 10.7554/eLife.71103 35060903 PMC8824465

[B85] Hamilton L. S. Sohl-Dickstein J. Huth A. G. Carels V. M. Deisseroth K. Bao S. (2013). Optogenetic activation of an inhibitory network enhances feedforward functional connectivity in auditory cortex. *Neuron* 80 1066–1076. 10.1016/j.neuron.2013.08.017 24267655 PMC3841078

[B86] Hanover J. L. Huang Z. J. Tonegawa S. Stryker M. P. (1999). Brain-derived neurotrophic factor overexpression induces precocious critical period in mouse visual cortex. *J. Neurosci.* 19:RC40. 10.1523/JNEUROSCI.19-22-j0003.1999 10559430 PMC2424259

[B87] Happel M. F. K. Niekisch H. Rivera L. L. C. Ohl F. W. Deliano M. Frischknecht R. (2014). Enhanced cognitive flexibility in reversal learning induced by removal of the extracellular matrix in auditory cortex. *Proc. Natl. Acad. Sci. U.S.A.* 111 2800–2805. 10.1073/pnas.1310272111 24550310 PMC3932855

[B88] Harauzov A. Spolidoro M. DiCristo G. De Pasquale R. Cancedda L. Pizzorusso T. (2010). Reducing intracortical inhibition in the adult visual cortex promotes ocular dominance plasticity. *J. Neurosci.* 30 361–371. 10.1523/JNEUROSCI.2233-09.2010 20053917 PMC6632513

[B89] Härtig W. Derouiche A. Welt K. Brauer K. Grosche J. Mäder M. (1999). Cortical neurons immunoreactive for the potassium channel Kv3.1b subunit are predominantly surrounded by perineuronal nets presumed as a buffering system for cations. *Brain Res.* 842 15–29. 10.1016/S0006-8993(99)01784-9 10526091

[B90] Headley D. B. Weinberger N. M. (2011). Gamma-band activation predicts both associative memory and cortical plasticity. *J. Neurosci.* 31 12748–12758. 10.1523/JNEUROSCI.2528-11.2011 21900554 PMC3180928

[B91] Heiss J. Katz Y. Ganmor E. Lampl I. (2009). Shift in the balance between excitation and inhibition during sensory adaptation of S1 neurons. *J. Neurosci.* 28 13320–13330. 10.1523/JNEUROSCI.2646-08.2008 19052224 PMC6671605

[B92] Hensch T. K. (2004). Critical period regulation. *Annu. Rev. Neurosci.* 27 549–579. 10.1146/annurev.neuro.27.070203.144327 15217343

[B93] Hensch T. K. Fagiolini M. Mataga N. Stryker M. P. Baekkeskov S. Kash S. F. (1998). Local GABA circuit control of experience-dependent plasticity in developing visual cortex. *Science* 282 1504–1508. 10.1126/science.282.5393.1504 9822384 PMC2851625

[B94] Hofer S. B. Ko H. Pichler B. Vogelstein J. Ros H. Zeng H. (2011). Differential connectivity and response dynamics of excitatory and inhibitory neurons in visual cortex. *Nat. Neurosci.* 14 1045–1052. 10.1038/nn.2876 21765421 PMC6370002

[B95] Hofer S. B. Mrsic-Flogel T. D. Bonhoeffer T. Hübener M. (2006). Prior experience enhances plasticity in adult visual cortex. *Nat. Neurosci.* 9 127–132. 10.1038/nn1610 16327785

[B96] Hou X. Yoshioka N. Tsukano H. Sakai A. Miyata S. Watanabe Y. (2017). Chondroitin sulfate is required for onset and offset of critical period plasticity in visual cortex. *Sci. Rep.* 7:12646. 10.1038/s41598-017-04007-x 28974755 PMC5626782

[B97] Huang X. Stodieck S. K. Goetze B. Cui L. Wong M. H. Wenzel C. (2015). Progressive maturation of silent synapses governs the duration of a critical period. *Proc. Natl. Acad. Sci. U.S.A.* 112 E3131–E3140. 10.1073/pnas.1506488112 26015564 PMC4475980

[B98] Huang Z. J. (2014). Toward a genetic dissection of cortical circuits in the mouse. *Neuron* 83 1284–1302. 10.1016/j.neuron.2014.08.041 25233312 PMC4169123

[B99] Huang Z. J. Kirkwood A. Pizzorusso T. Porciatti V. Morales B. Bear M. F. (1999). BDNF regulates the maturation of inhibition and the critical period of plasticity in mouse visual cortex. *Cell* 98 739–755. 10.1016/S0092-8674(00)81509-3 10499792

[B100] Hubel D. H. Wiesel T. N. (1963). Shape and arrangement of columns in cat’s striate cortex. *J. Physiol.* 165 559–568.2. 10.1113/jphysiol.1963.sp007079 13955384 PMC1359325

[B101] Iny K. Heynen A. J. Sklar E. Bear M. F. (2006). Bidirectional modifications of visual acuity induced by monocular deprivation in juvenile and adult rats. *J. Neurosci.* 26 7368–7374. 10.1523/JNEUROSCI.0124-06.2006 16837583 PMC6674195

[B102] Iwai Y. Fagiolini M. Obata K. Hensch T. K. (2003). Rapid critical period induction by tonic inhibition in visual cortex. *J. Neurosci.* 23 6695–6702. 10.1523/JNEUROSCI.23-17-06695.2003 12890762 PMC6740711

[B103] Jiang B. Huang Z. J. Morales B. Kirkwood A. (2005). Maturation of GABAergic transmission and the timing of plasticity in visual cortex. *Brain Res. Rev.* 50 126–133. 10.1016/j.brainresrev.2005.05.007 16024085

[B104] Jiang H. H. Guo A. Chiu A. Li H. Lai C. S. W. Lau C. G. (2021). Target-specific control of piriform cortical output via distinct inhibitory circuits. *FASEB J.* 35:e21944. 10.1096/fj.202100757R 34569087 PMC12316094

[B105] Jonas P. Racca C. Sakmann B. Seeburg P. H. Monyer H. (1994). Differences in Ca^2+^ permeability of AMPA-type glutamate receptor channels in neocortical neurons caused by differential GluR-B subunit expression. *Neuron* 12 1281–1289. 10.1016/0896-6273(94)90444-8 8011338

[B106] Kalemaki K. Konstantoudaki X. Tivodar S. Sidiropoulou K. Karagogeos D. (2018). Mice with decreased number of interneurons exhibit aberrant spontaneous and oscillatory activity in the cortex. *Front. Neural Circuits* 12:96. 10.3389/fncir.2018.00096 30429776 PMC6220423

[B107] Kamphuis W. Huisman E. Wadman W. J. Heizmann C. W. Lopes da Silva F. H. (1989). Kindling induced changes in parvalbumin immunoreactivity in rat hippocampus and its relation to long-term decrease in GABA-immunoreactivity. *Brain Res.* 479 23–34. 10.1016/0006-8993(89)91331-0 2924151

[B108] Kaplan E. S. Cooke S. F. Komorowski R. W. Chubykin A. A. Thomazeau A. Khibnik L. A. (2016). Contrasting roles for parvalbumin-expressing inhibitory neurons in two forms of adult visual cortical plasticity. *eLife* 5:e11450. 10.7554/eLife.11450 26943618 PMC4786407

[B109] Karnani M. M. Agetsuma M. Yuste R. (2014). A blanket of inhibition: functional inferences from dense inhibitory connectivity. *Curr. Opin. Neurobiol.* 26 96–102. 10.1016/j.conb.2013.12.015 24440415 PMC4024353

[B110] Kawaguchi Y. (1995). Physiological subgroups of nonpyramidal cells with specific morphological characteristics in layer II/III of rat frontal cortex. *J. Neurosci.* 15 2638–2655. 10.1523/JNEUROSCI.15-04-02638.1995 7722619 PMC6577784

[B111] Kawaguchi Y. Hama K. (1987). Two subtypes of non-pyramidal cells in rat hippocampal formation identified by intracellular recording and HRP injection. *Brain Res.* 411 190–195. 10.1016/0006-8993(87)90700-1 3607424

[B112] Kawaguchi Y. Kubota Y. (1993). Correlation of physiological subgroupings of nonpyramidal cells with parvalbumin- and calbindinD28k-immunoreactive neurons in layer V of rat frontal cortex. *J. Neurophysiol.* 70 387–396. 10.1152/jn.1993.70.1.387 8395585

[B113] Kawaguchi Y. Otsuka T. Morishima M. Ushimaru M. Kubota Y. (2019). Control of excitatory hierarchical circuits by parvalbumin-FS basket cells in layer 5 of the frontal cortex: insights for cortical oscillations. *J. Neurophysiol.* 121, 2222–2236. 10.1152/jn.00778.2018 30995139 PMC6620693

[B114] Keller C. H. Kaylegian K. Wehr M. (2018). Gap encoding by parvalbumin-expressing interneurons in auditory cortex. *J. Neurophysiol.* 120 105–114. 10.1152/jn.00911.2017 29589814 PMC6093948

[B115] Kerlin A. M. Andermann M. L. Berezovskii V. K. Reid R. C. (2010). Broadly tuned response properties of diverse inhibitory neuron subtypes in mouse visual cortex. *Neuron* 67 858–871. 10.1016/j.neuron.2010.08.002 20826316 PMC3327881

[B116] Kim H. Ährlund-Richter S. Wang X. Deisseroth K. Carlén M. (2016). Prefrontal parvalbumin neurons in control of attention. *Cell* 164 208–218. 10.1016/j.cell.2015.11.038 26771492 PMC4715187

[B117] Kim T. Thankachan S. McKenna J. T. McNally J. M. Yang C. Choi J. H. (2015). Cortically projecting basal forebrain parvalbumin neurons regulate cortical gamma band oscillations. *Proc. Natl. Acad. Sci. U.S.A.* 112 3535–3540. 10.1073/pnas.1413625112 25733878 PMC4371918

[B118] Kissinger S. T. Pak A. Tang Y. Masmanidis S. C. Chubykin A. A. (2018). Oscillatory encoding of visual stimulus familiarity. *J. Neurosci.* 38 6223–6240. 10.1523/JNEUROSCI.3646-17.2018 29915138 PMC6031580

[B119] Kissinger S. T. Wu Q. Quinn C. J. Anderson A. K. Pak A. Chubykin A. A. (2020). Visual experience-dependent oscillations and underlying circuit connectivity changes are impaired in Fmr1 KO mice. *Cell Rep.* 31:107486. 10.1016/j.celrep.2020.03.050 32268079 PMC7201849

[B120] Klampfl S. Maass W. (2013). Emergence of dynamic memory traces in cortical microcircuit models through STDP. *J. Neurosci.* 33 11515–11529. 10.1523/JNEUROSCI.5044-12.2013 23843522 PMC6618695

[B121] Korotkova T. Fuchs E. C. Ponomarenko A. von Engelhardt J. Monyer H. (2010). NMDA receptor ablation on parvalbumin-positive interneurons impairs hippocampal synchrony, spatial representations, and working memory. *Neuron* 68 557–569. 10.1016/j.neuron.2010.09.017 21040854

[B122] Krishnan K. Lau B. Y. B. Ewall G. Huang Z. J. Shea S. D. (2017). MECP2 regulates cortical plasticity underlying a learned behaviour in adult female mice. *Nat. Commun.* 8:14077. 10.1038/ncomms14077 28098153 PMC5253927

[B123] Krishnan K. Wang B.-S. Lu J. Wang L. Maffei A. Cang J. (2015). MeCP2 regulates the timing of critical period plasticity that shapes functional connectivity in primary visual cortex. *Proc. Natl. Acad. Sci. U.S.A.* 112 E4782–E4791. 10.1073/pnas.1506499112 26261347 PMC4553776

[B124] Kuhlman S. J. Lu J. Lazarus M. S. Huang Z. J. (2010). Maturation of GABAergic inhibition promotes strengthening of temporally coherent inputs among convergent pathways. *PLoS Comput. Biol.* 6:e1000797. 10.1371/journal.pcbi.1000797 20532211 PMC2880567

[B125] Kuhlman S. J. Olivas N. D. Tring E. Ikrar T. Xu X. Trachtenberg J. T. (2013). A disinhibitory microcircuit initiates critical-period plasticity in the visual cortex. *Nature* 501 543–546. 10.1038/nature12485 23975100 PMC3962838

[B126] Kuhlman S. J. Tring E. Trachtenberg J. T. (2011). Fast-spiking interneurons have an initial orientation bias that is lost with vision. *Nat. Neurosci.* 14 1121–1123. 10.1038/nn.2890 21750548 PMC3164933

[B127] Lander C. Kind P. Maleski M. Hockfield S. (1997). A family of activity-dependent neuronal cell-surface chondroitin sulfate proteoglycans in cat visual cortex. *J. Neurosci.* 17 1928–1939. 10.1523/JNEUROSCI.17-06-01928.1997 9045722 PMC6793771

[B128] Large A. M. Vogler N. W. Canto-Bustos M. Friason F. K. Schick P. Oswald A.-M. M. (2018). Differential inhibition of pyramidal cells and inhibitory interneurons along the rostrocaudal axis of anterior piriform cortex. *Proc. Natl. Acad. Sci. U.S.A.* 115 E8067–E8076. 10.1073/pnas.1802428115 30087186 PMC6112735

[B129] Lau B. Y. B. Krishnan K. Huang Z. J. Shea S. D. (2020). Maternal experience-dependent cortical plasticity in mice is circuit- and stimulus-specific and requires MECP2. *J. Neurosci.* 40 1514–1526. 10.1523/JNEUROSCI.1964-19.2019 31911459 PMC7044728

[B130] Lee S.-H. Kwan A. C. Zhang S. Phoumthipphavong V. Flannery J. G. Masmanidis S. C. (2012). Activation of specific interneurons improves V1 feature selectivity and visual perception. *Nature* 488 379–383. 10.1038/nature11312 22878719 PMC3422431

[B131] LeMessurier A. M. Feldman D. E. (2018). Plasticity of population coding in primary sensory cortex. *Curr. Opin. Neurobiol.* 53 50–56. 10.1016/j.conb.2018.04.029 29775823 PMC7211502

[B132] Lensjø K. K. Lepperød M. E. Dick G. Hafting T. Fyhn M. (2017). Removal of perineuronal nets unlocks juvenile plasticity through network mechanisms of decreased inhibition and increased gamma activity. *J. Neurosci.* 37 1269–1283. 10.1523/JNEUROSCI.2504-16.2016 28039374 PMC6596863

[B133] Levay S. Stryker M. P. Shatz C. J. (1978). Ocular dominance columns and their development in layer IV of the cat’s visual cortex: a quantitative study. *J. Comp. Neurol.* 179, 223–244. 10.1002/cne.901790113 8980725

[B134] Li H. Wang J. Liu G. Xu J. Huang W. Song C. (2021). Phasic off responses of auditory cortex underlie perception of sound duration. *Cell Rep.* 35:109003. 10.1016/j.celrep.2021.109003 33882311 PMC8154544

[B135] Li L. Xiong X. R. Ibrahim L. A. Yuan W. Tao H. W. Zhang L. I. (2015). Differential receptive field properties of parvalbumin and somatostatin inhibitory neurons in mouse auditory cortex. *Cereb. Cortex* 25 1782–1791. 10.1093/cercor/bht417 24425250 PMC4459283

[B136] Li Y.-T. Ma W.-P. Li L.-Y. Ibrahim L. A. Wang S.-Z. Tao H. W. (2012). Broadening of inhibitory tuning underlies contrast-dependent sharpening of orientation selectivity in mouse visual cortex. *J. Neurosci.* 32 16466–16477. 10.1523/JNEUROSCI.3221-12.2012 23152629 PMC3548445

[B137] Lin F. G. Galindo-Leon E. E. Ivanova T. N. Mappus R. C. Liu R. C. (2013). A role for maternal physiological state in preserving auditory cortical plasticity for salient infant calls. *Neuroscience* 247 102–116. 10.1016/j.neuroscience.2013.05.020 23707982 PMC3722272

[B138] Lintas A. Sánchez-Campusano R. Villa A. E. P. Gruart A. Delgado-García J. M. (2021). Operant conditioning deficits and modified local field potential activities in parvalbumin-deficient mice. *Sci. Rep.* 11:2970. 10.1038/s41598-021-82519-3 33536607 PMC7859233

[B139] Liu R. C. Schreiner C. E. (2007). Auditory cortical detection and discrimination correlates with communicative significance. *PLoS Biol.* 5:e173. 10.1371/journal.pbio.0050173 17564499 PMC1891324

[B140] Lu J. Li C. Zhao J.-P. Poo M. Zhang X. (2007). Spike-timing-dependent plasticity of neocortical excitatory synapses on inhibitory interneurons depends on target cell type. *J. Neurosci.* 27 9711–9720. 10.1523/JNEUROSCI.2513-07.2007 17804631 PMC6672961

[B141] Ma W.-P. Liu B.-H. Li Y.-T. Josh Huang Z. Zhang L. I. Tao H. W. (2010). Visual representations by cortical somatostatin inhibitory neurons–selective but with weak and delayed responses. *J. Neurosci.* 30 14371–14379. 10.1523/JNEUROSCI.3248-10.2010 20980594 PMC3001391

[B142] Maffei A. Lambo M. E. Turrigiano G. G. (2010). Critical period for inhibitory plasticity in RodentBinocular V1. *J. Neurosci.* 30 3304–3309. 10.1523/JNEUROSCI.5340-09.2010 20203190 PMC2848504

[B143] Maffei A. Nataraj K. Nelson S. B. Turrigiano G. G. (2006). Potentiation of cortical inhibition by visual deprivation. *Nature* 443 81–84. 10.1038/nature05079 16929304

[B144] Magnowska M. Gorkiewicz T. Suska A. Wawrzyniak M. Rutkowska-Wlodarczyk I. Kaczmarek L. (2016). Transient ECM protease activity promotes synaptic plasticity. *Sci. Rep.* 6:27757. 10.1038/srep27757 27282248 PMC4901294

[B145] Markram H. Toledo-Rodriguez M. Wang Y. Gupta A. Silberberg G. Wu C. (2004). Interneurons of the neocortical inhibitory system. *Nat. Rev. Neurosci.* 5 793–807. 10.1038/nrn1519 15378039

[B146] Marlin B. J. Mitre M. D’Amour J. A. Chao M. V. Froemke R. C. (2015). Oxytocin enables maternal behaviour by balancing cortical inhibition. *Nature* 520 499–504. 10.1038/nature14402 25874674 PMC4409554

[B147] McGee A. W. Yang Y. Fischer Q. S. Daw N. W. Strittmatter S. M. (2005). Experience-driven plasticity of visual cortex limited by myelin and nogo receptor. *Science* 309 2222–2226. 10.1126/science.1114362 16195464 PMC2856689

[B148] McGirr A. LeDue J. Chan A. W. Boyd J. D. Metzak P. D. Murphy T. H. (2020). Stress impacts sensory variability through cortical sensory activity motifs. *Transl. Psychiatry* 10:20. 10.1038/s41398-020-0713-1 32066714 PMC7026117

[B149] McRae P. A. Rocco M. M. Kelly G. Brumberg J. C. Matthews R. T. (2007). Sensory deprivation alters aggrecan and perineuronal net expression in the mouse barrel cortex. *J. Neurosci.* 27 5405–5413. 10.1523/JNEUROSCI.5425-06.2007 17507562 PMC6672348

[B150] Meliza C. D. Dan Y. (2006). Receptive-field modification in rat visual cortex induced by paired visual stimulation and single-cell spiking. *Neuron* 49 183–189. 10.1016/j.neuron.2005.12.009 16423693

[B151] Mittmann W. Koch U. Häusser M. (2005). Feed-forward inhibition shapes the spike output of cerebellar Purkinje cells. *J. Physiol.* 563 369–378. 10.1113/jphysiol.2004.075028 15613376 PMC1665592

[B152] Miyata S. Nadanaka S. Igarashi M. Kitagawa H. (2018). Structural variation of chondroitin sulfate chains contributes to the molecular heterogeneity of perineuronal nets. *Front. Integr. Neurosci.* 2:3. 10.3389/fnint.2018.00003 29456495 PMC5801575

[B153] Moore A. K. Wehr M. (2013). Parvalbumin-expressing inhibitory interneurons in auditory cortex are well-tuned for frequency. *J. Neurosci.* 33 13713–13723. 10.1523/JNEUROSCI.0663-13.2013 23966693 PMC3755717

[B154] Moreno-López Y. Hollis E. R. (2021). Sensory circuit remodeling and movement recovery after spinal cord injury. *Front. Neurosci.* 15:787690. 10.3389/fnins.2021.787690 34955735 PMC8692650

[B155] Muehlhan M. Alexander N. Trautmann S. Weckesser L. J. Vogel S. Kirschbaum C. (2020). Cortisol secretion predicts functional macro-scale connectivity of the visual cortex: a data-driven Multivoxel Pattern Analysis (MVPA). *Psychoneuroendocrinology* 117:104695. 10.1016/j.psyneuen.2020.104695 32361170

[B156] Murase S. Lantz C. L. Quinlan E. M. (2017). Light reintroduction after dark exposure reactivates plasticity in adults via perisynaptic activation of MMP-9. *eLife* 6:e27345. 10.7554/eLife.27345 28875930 PMC5630258

[B157] Nahmani M. Turrigiano G. G. (2014). Adult cortical plasticity following injury: recapitulation of critical period mechanisms? *Neuroscience* 283 4–16. 10.1016/j.neuroscience.2014.04.029 24791715 PMC4216647

[B158] Natan R. G. Briguglio J. J. Mwilambwe-Tshilobo L. Jones S. I. Aizenberg M. Goldberg E. M. (2015). Complementary control of sensory adaptation by two types of cortical interneurons. *eLife* 4:e09868. 10.7554/eLife.09868 26460542 PMC4641469

[B159] Niell C. M. Stryker M. P. (2010). Modulation of visual responses by behavioral state in mouse visual cortex. *Neuron* 65 472–479. 10.1016/j.neuron.2010.01.033 20188652 PMC3184003

[B160] Nowicka D. Soulsby S. Skangiel-Kramska J. Glazewski S. (2009). Parvalbumin-containing neurons, perineuronal nets and experience-dependent plasticity in murine barrel cortex. *Eur. J. Neurosci.* 30 2053–2063. 10.1111/j.1460-9568.2009.06996.x 20128844

[B161] Packer A. M. Yuste R. (2011). Dense, unspecific connectivity of neocortical parvalbumin-positive interneurons: a canonical microcircuit for inhibition? *J. Neurosci.* 31 13260–13271. 10.1523/JNEUROSCI.3131-11.2011 21917809 PMC3178964

[B162] Pakan J. M. Lowe S. C. Dylda E. Keemink S. W. Currie S. P. Coutts C. A. (2016). Behavioral-state modulation of inhibition is context-dependent and cell type specific in mouse visual cortex. *eLife* 5:e14985. 10.7554/eLife.14985 27552056 PMC5030095

[B163] Patz S. Grabert J. Gorba T. Wirth M. J. Wahle P. (2004). Parvalbumin expression in visual cortical interneurons depends on neuronal activity and TrkB ligands during an early period of postnatal development. *Cereb. Cortex* 14 342–351. 10.1093/cercor/bhg132 14754872

[B164] Pérez-González D. Malmierca M. S. (2012). Variability of the time course of stimulus-specific adaptation in the inferior colliculus. *Front. Neural Circuits* 6:107. 10.3389/fncir.2012.00107 23293586 PMC3530767

[B165] Petilla Interneuron Nomenclature Group, Ascoli G. A. Alonso-Nanclares L. Anderson S. A. Barrionuevo G. Benavides-Piccione R. (2008). Petilla terminology: nomenclature of features of GABAergic interneurons of the cerebral cortex. *Nat. Rev. Neurosci.* 9 557–568. 10.1038/nrn2402 18568015 PMC2868386

[B166] Pielecka-Fortuna J. Kalogeraki E. Fortuna M. G. Löwel S. (2015). Optimal level activity of matrix metalloproteinases is critical for adult visual plasticity in the healthy and stroke-affected brain. *eLife* 4:e11290. 10.7554/eLife.11290 26609811 PMC4718812

[B167] Pirbhoy P. S. Rais M. Lovelace J. W. Woodard W. Razak K. A. Binder D. K. (2020). Acute pharmacological inhibition of matrix metalloproteinase-9 activity during development restores perineuronal net formation and normalizes auditory processing in *Fmr1* KO mice. *J. Neurochem.* 155 538–558. 10.1111/jnc.15037 32374912 PMC7644613

[B168] Pizzorusso T. Medini P. Berardi N. Chierzi S. Fawcett J. W. Maffei L. (2002). Reactivation of ocular dominance plasticity in the adult visual cortex. *Science* 298 1248–1251. 10.1126/science.1072699 12424383

[B169] Pluta S. Naka A. Veit J. Telian G. Yao L. Hakim R. (2015). A direct translaminar inhibitory circuit tunes cortical output. *Nat. Neurosci.* 18 1631–1640. 10.1038/nn.4123 26414615 PMC4624464

[B170] Poo C. Isaacson J. S. (2009). Odor representations in olfactory cortex: “sparse” coding, global inhibition, and oscillations. *Neuron* 62 850–861. 10.1016/j.neuron.2009.05.022 19555653 PMC2702531

[B171] Pouchelon G. Dwivedi D. Bollmann Y. Agba C. K. Xu Q. Mirow A. M. C. (2021). The organization and development of cortical interneuron presynaptic circuits are area specific. *Cell Rep.* 37:109993. 10.1016/j.celrep.2021.109993 34758329 PMC8832360

[B172] Ray S. Maunsell J. H. R. (2015). Do gamma oscillations play a role in cerebral cortex? *Trends Cogn. Sci.* 19 78–85. 10.1016/j.tics.2014.12.002 25555444 PMC5403517

[B173] Reimers S. Hartlage-Rübsamen M. Brückner G. Roßner S. (2007). Formation of perineuronal nets in organotypic mouse brain slice cultures is independent of neuronal glutamatergic activity. *Eur. J. Neurosci.* 25 2640–2648. 10.1111/j.1460-9568.2007.05514.x 17561838

[B174] Ribic A. (2020). Stability in the face of change: lifelong experience-dependent plasticity in the sensory cortex. *Front. Cell. Neurosci.* 14:76. 10.3389/fncel.2020.00076 32372915 PMC7186337

[B175] Rikhye R. V. Yildirim M. Hu M. Breton-Provencher V. Sur M. (2021). Reliable sensory processing in mouse visual cortex through cooperative interactions between somatostatin and parvalbumin interneurons. *J. Neurosci.* 41 8761–8778. 10.1523/JNEUROSCI.3176-20.2021 34493543 PMC8528503

[B176] Rodarie D. Verasztó C. Roussel Y. Reimann M. Keller D. Ramaswamy S. (2021). A method to estimate the cellular composition of the mouse brain from heterogeneous datasets. *bioRxiv* [Preprint]. 10.1101/2021.11.20.469384 PMC9838873 36542673

[B177] Roll L. Faissner A. (2014). Influence of the extracellular matrix on endogenous and transplanted stem cells after brain damage. *Front. Cell. Neurosci.* 8:219. 10.3389/fncel.2014.00219 25191223 PMC4137450

[B178] Rosenblatt J. S. (1967). Nonhormonal basis of maternal behavior in the rat. *Science* 156 1512–1514. 10.1126/science.156.3781.1512 5611028

[B179] Rossier J. Bernard A. Cabungcal J.-H. Perrenoud Q. Savoye A. Gallopin T. (2015). Cortical fast-spiking parvalbumin interneurons enwrapped in the perineuronal net express the metallopeptidases Adamts8, Adamts15 and Neprilysin. *Mol. Psychiatry* 20 154–161. 10.1038/mp.2014.162 25510509 PMC4356748

[B180] Rowland J. M. van der Plas T. L. Loidolt M. Lees R. M. Keeling J. Dehning J. (2021). Perception and propagation of activity through the cortical hierarchy is determined by neural variability. *bioRxiv* [Preprint]. 10.1101/2021.12.28.474343 PMC10471496 37640911

[B181] Rubenstein J. L. R. Merzenich M. M. (2003). Model of autism: increased ratio of excitation/inhibition in key neural systems. *Genes Brain Behav.* 2 255–267. 10.1034/j.1601-183x.2003.00037.x 14606691 PMC6748642

[B182] Rudy B. Fishell G. Lee S. Hjerling-Leffler J. (2011). Three groups of interneurons account for nearly 100% of neocortical GABAergic neurons. *Dev. Neurobiol.* 71 45–61. 10.1002/dneu.20853 21154909 PMC3556905

[B183] Rudy B. McBain C. J. (2001). Kv3 channels: voltage-gated K^+^ channels designed for high-frequency repetitive firing. *Trends Neurosci.* 24 517–526. 10.1016/s0166-2236(00)01892-0 11506885

[B184] Runyan C. A. Schummers J. Van Wart A. Kuhlman S. J. Wilson N. R. Huang Z. J. (2010). Response features of parvalbumin-expressing interneurons suggest precise roles for subtypes of inhibition in visual cortex. *Neuron* 67 847–857. 10.1016/j.neuron.2010.08.006 20826315 PMC2948796

[B185] Saiepour M. H. Rajendran R. Omrani A. Ma W.-P. Tao H. W. Heimel J. A. (2015). Ocular dominance plasticity disrupts binocular inhibition-excitation matching in visual cortex. *Curr. Biol.* 25 713–721. 10.1016/j.cub.2015.01.024 25754642 PMC4464670

[B186] Sale A. Maya Vetencourt J. F. Medini P. Cenni M. C. Baroncelli L. De Pasquale R. (2007). Environmental enrichment in adulthood promotes amblyopia recovery through a reduction of intracortical inhibition. *Nat. Neurosci.* 10 679–681. 10.1038/nn1899 17468749

[B187] Sato M. Stryker M. P. (2008). Distinctive features of adult ocular dominance plasticity. *J. Neurosci.* 28 10278–10286. 10.1523/JNEUROSCI.2451-08.2008 18842887 PMC2851628

[B188] Sawtell N. B. Frenkel M. Y. Philpot B. D. Nakazawa K. Tonegawa S. Bear M. F. (2003). Nmda receptor-dependent ocular dominance plasticity in adult visual cortex. *Neuron* 38 977–985. 10.1016/S0896-6273(03)00323-4 12818182

[B189] Schwaller B. (2012). The use of transgenic mouse models to reveal the functions of Ca^2+^ buffer proteins in excitable cells. *Biochim. Biophys. Acta Gen. Subj.* 1820 1294–1303. 10.1016/j.bbagen.2011.11.008 22138448

[B190] Severin D. Hong S. Z. Roh S.-E. Huang S. Zhou J. Bridi M. C. D. (2021). All-or-none disconnection of pyramidal inputs onto parvalbumin-positive interneurons gates ocular dominance plasticity. *Proc. Natl. Acad. Sci. U.S.A.* 118 e2105388118. 10.1073/pnas.2105388118 34508001 PMC8449314

[B191] Sewell G. D. (1970). Ultrasonic communication in rodents. *Nature* 227 410–410. 10.1038/227410a0 5464098

[B192] Shadlen M. N. Newsome W. T. (1994). Noise, neural codes and cortical organization. *Curr. Opin. Neurobiol.* 4 569–579. 10.1016/0959-4388(94)90059-0 7812147

[B193] Shepard K. N. Lin F. G. Zhao C. L. Chong K. K. Liu R. C. (2015). Behavioral relevance helps untangle natural vocal categories in a specific subset of core auditory cortical pyramidal neurons. *J. Neurosci.* 35 2636–2645. 10.1523/JNEUROSCI.3803-14.2015 25673855 PMC4323536

[B194] Shin H. Byun J. Roh D. Choi N. Shin H.-S. Cho I.-J. (2022). Interference-free, lightweight wireless neural probe system for investigating brain activity during natural competition. *Biosens. Bioelectron.* 195:113665. 10.1016/j.bios.2021.113665 34610533

[B195] Smith G. B. Heynen A. J. Bear M. F. (2009). Bidirectional synaptic mechanisms of ocular dominance plasticity in visual cortex. *Philos. Trans. R. Soc. B Biol. Sci.* 364 357–367. 10.1098/rstb.2008.0198 18977732 PMC2674473

[B196] Sobolewski A. Swiejkowski D. A. Wróbel A. Kublik E. (2011). The 5–12Hz oscillations in the barrel cortex of awake rats – sustained attention during behavioral idling? *Clin. Neurophysiol.* 122 483–489. 10.1016/j.clinph.2010.08.006 20826110

[B197] Sohal V. S. Zhang F. Yizhar O. Deisseroth K. (2009). Parvalbumin neurons and gamma rhythms enhance cortical circuit performance. *Nature* 459 698–702. 10.1038/nature07991 19396159 PMC3969859

[B198] Sommeijer J.-P. Ahmadlou M. Saiepour M. H. Seignette K. Min R. Heimel J. A. (2017). Thalamic inhibition regulates critical-period plasticity in visual cortex and thalamus. *Nat. Neurosci.* 20 1715–1721. 10.1038/s41593-017-0002-3 29184199

[B199] Southwell D. G. Froemke R. C. Alvarez-Buylla A. Stryker M. P. Gandhi S. P. (2010). Cortical plasticity induced by inhibitory neuron transplantation. *Science* 327 1145–1148. 10.1126/science.1183962 20185728 PMC3164148

[B200] Stolzenberg D. S. Champagne F. A. (2016). Hormonal and non-hormonal bases of maternal behavior: the role of experience and epigenetic mechanisms. *Horm. Behav.* 77 204–210. 10.1016/j.yhbeh.2015.07.005 26172856

[B201] Sugiyama S. Di Nardo A. A. Aizawa S. Matsuo I. Volovitch M. Prochiantz A. (2008). Experience-dependent transfer of Otx2 homeoprotein into the visual cortex activates postnatal plasticity. *Cell* 134 508–520. 10.1016/j.cell.2008.05.054 18692473

[B202] Taborsky M. Hofmann H. A. Beery A. K. Blumstein D. T. Hayes L. D. Lacey E. A. (2015). Taxon matters: promoting integrative studies of social behavior: NESCent Working Group on integrative models of vertebrate sociality: evolution, mechanisms, and emergent properties. *Trends Neurosci.* 38 189–191. 10.1016/j.tins.2015.01.004 25656466

[B203] Takatsuru Y. Koibuchi N. (2015). Alteration of somatosensory response in adulthood by early life stress. *Front. Mol. Neurosci.* 8:15. 10.3389/fnmol.2015.00015 26041988 PMC4436820

[B204] Tamamaki N. Yanagawa Y. Tomioka R. Miyazaki J.-I. Obata K. Kaneko T. (2003). Green fluorescent protein expression and colocalization with calretinin, parvalbumin, and somatostatin in the GAD67-GFP knock-in mouse. *J. Comp. Neurol.* 467 60–79. 10.1002/cne.10905 14574680

[B205] Tamás G. Buhl E. H. Lörincz A. Somogyi P. (2000). Proximally targeted GABAergic synapses and gap junctions synchronize cortical interneurons. *Nat. Neurosci.* 3 366–371. 10.1038/73936 10725926

[B206] Tang Y. Stryker M. P. Alvarez-Buylla A. Espinosa J. S. (2014). Cortical plasticity induced by transplantation of embryonic somatostatin or parvalbumin interneurons. *Proc. Natl. Acad. Sci. U.S.A.* 111 18339–18344. 10.1073/pnas.1421844112 25489113 PMC4280644

[B207] Tasic B. (2018). Single cell transcriptomics in neuroscience: cell classification and beyond. *Curr. Opin. Neurobiol.* 50 242–249. 10.1016/j.conb.2018.04.021 29738987

[B208] Trachtenberg J. T. (2015). Competition, inhibition, and critical periods of cortical plasticity. *Curr. Opin. Neurobiol.* 35 44–48. 10.1016/j.conb.2015.06.006 26126153 PMC5479701

[B209] Vecchia D. Beltramo R. Vallone F. Chéreau R. Forli A. Molano-Mazón M. (2020). Temporal sharpening of sensory responses by layer v in the mouse primary somatosensory cortex. *Curr. Biol.* 30 1589–1599.e10. 10.1016/j.cub.2020.02.004 32169206 PMC7198976

[B210] Vickers E. D. Clark C. Osypenko D. Fratzl A. Kochubey O. Bettler B. (2018). Parvalbumin-interneuron output synapses show spike-timing-dependent plasticity that contributes to auditory map remodeling. *Neuron* 99 720–735.e6. 10.1016/j.neuron.2018.07.018 30078579

[B211] Vinck M. Batista-Brito R. Knoblich U. Cardin J. A. (2015). Arousal and locomotion make distinct contributions to cortical activity patterns and visual encoding. *Neuron* 86 740–754. 10.1016/j.neuron.2015.03.028 25892300 PMC4425590

[B212] Volman V. Behrens M. M. Sejnowski T. J. (2011). Downregulation of parvalbumin at cortical GABA synapses reduces network gamma oscillatory activity. *J. Neurosci.* 31 18137–18148. 10.1523/JNEUROSCI.3041-11.2011 22159125 PMC3257321

[B213] Wegner F. Härtig W. Bringmann A. Grosche J. Wohlfarth K. Zuschratter W. (2003). Diffuse perineuronal nets and modified pyramidal cells immunoreactive for glutamate and the GABA(A) receptor alpha1 subunit form a unique entity in rat cerebral cortex. *Exp. Neurol.* 184 705–714. 10.1016/S0014-4886(03)00313-3 14769362

[B214] Wehr M. Zador A. M. (2003). Balanced inhibition underlies tuning and sharpens spike timing in auditory cortex. *Nature* 426 442–446. 10.1038/nature02116 14647382

[B215] Weible A. P. Liu C. Niell C. M. Wehr M. (2014). Auditory cortex is required for fear potentiation of gap detection. *J. Neurosci.* 34 15437–15445. 10.1523/JNEUROSCI.3408-14.2014 25392510 PMC4228141

[B216] Weinberger N. M. Miasnikov A. A. Bieszczad K. M. Chen J. C. (2013). Gamma band plasticity in sensory cortex is a signature of the strongest memory rather than memory of the training stimulus. *Neurobiol. Learn. Mem.* 104 49–63. 10.1016/j.nlm.2013.05.001 23669065 PMC3742680

[B217] Wen T. H. Afroz S. Reinhard S. M. Palacios A. R. Tapia K. Binder D. K. (2018). Genetic reduction of matrix metalloproteinase-9 promotes formation of perineuronal nets around parvalbumin-expressing interneurons and normalizes auditory cortex responses in developing fmr1 knock-out mice. *Cereb. Cortex* 28 3951–3964. 10.1093/cercor/bhx258 29040407 PMC6188540

[B218] Wenisch O. G. Noll J. van Hemmen J. L. (2005). Spontaneously emerging direction selectivity maps in visual cortex through STDP. *Biol. Cybern.* 93 239–247. 10.1007/s00422-005-0006-z 16195915

[B219] Wiesel T. N. Hubel D. H. (1963). Single-cell responses in striate cortex of kittens deprived of vision in one eye. J. Neurophysiol. 26, 1003–1017. 10.1152/jn.1963.26.6.1003 14084161

[B220] Wilent W. B. Contreras D. (2005). Dynamics of excitation and inhibition underlying stimulus selectivity in rat somatosensory cortex. *Nat. Neurosci.* 8 1364–1370. 10.1038/nn1545 16158064

[B221] Wilson N. R. Runyan C. A. Wang F. L. Sur M. (2012). Division and subtraction by distinct cortical inhibitory networks in vivo. *Nature* 488 343–348. 10.1038/nature11347 22878717 PMC3653570

[B222] Wöhr M. Orduz D. Gregory P. Moreno H. Khan U. Vörckel K. J. (2015). Lack of parvalbumin in mice leads to behavioral deficits relevant to all human autism core symptoms and related neural morphofunctional abnormalities. *Transl. Psychiatry* 5:e525. 10.1038/tp.2015.19 25756808 PMC4354349

[B223] Womelsdorf T. Fries P. Mitra P. P. Desimone R. (2006). Gamma-band synchronization in visual cortex predicts speed of change detection. *Nature* 439 733–736. 10.1038/nature04258 16372022

[B224] Ye Q. Miao Q. (2013). Experience-dependent development of perineuronal nets and chondroitin sulfate proteoglycan receptors in mouse visual cortex. *Matrix Biol.* 32 352–363. 10.1016/j.matbio.2013.04.001 23597636

[B225] You Y. Gupta V. (2018). The extracellular matrix and remyelination strategies in multiple sclerosis. *eNeuro* 5:ENEURO.0435-17.2018. 10.1523/ENEURO.0435-17.2018 29662941 PMC5898789

[B226] Zariwala H. Madisen L. Ahrens K. Bernard A. Lein E. Jones A. (2011). Visual tuning properties of genetically identified layer 2/3 neuronal types in the primary visual cortex of cre-transgenic mice. *Front. Syst. Neurosci.* 4:162. 10.3389/fnsys.2010.00162 21283555 PMC3028542

[B227] Zhang L. I. Tan A. Y. Y. Schreiner C. E. Merzenich M. M. (2003). Topography and synaptic shaping of direction selectivity in primary auditory cortex. *Nature* 424 201–205. 10.1038/nature01796 12853959

[B228] Zhu Y. Qiao W. Liu K. Zhong H. Yao H. (2015). Control of response reliability by parvalbumin-expressing interneurons in visual cortex. *Nat. Commun.* 6:6802. 10.1038/ncomms7802 25869033

